# General disease factor: evidence of a unifying dimension across mental and physical illness in children and adolescents

**DOI:** 10.1136/bmjment-2025-301592

**Published:** 2025-06-03

**Authors:** Miguel Garcia-Argibay, Valerie Brandt, Hongyi Sun, Marco Solmi, Paul Lichtenstein, Henrik Larsson, Samuele Cortese

**Affiliations:** 1 Developmental EPI (Evidence synthesis, Prediction, Implementation) lab, Centre for Innovation in Mental Health, Faculty of Environmental and Life Sciences, University of Southampton, Southampton, UK; 2 Centre for Population Health, Research Department, Division for Mental Health, Haukeland University Hospital, Bergen, Norway; 3 School of Medical Sciences, Faculty of Medicine and Health, Örebro universitet, Orebro, Sweden; 4 Department of Medical Epidemiology and Biostatistics, Karolinska Institute, Stockholm, Sweden; 5 Clinic of Psychiatry, Social Psychiatry and Psychotherapy, Hannover Medical School, Hanover, Germany; 6 Department of Psychiatry, University of Ottawa, Ottawa, Ontario, Canada; 7 SCIENCES lab, Department of Mental Health, The Ottawa Hospital, Ottawa, Ontario, Canada; 8 Ottawa Hospital Research Institute Clinical Epidemiology Program, University of Ottawa, Ottawa, ON, Canada; 9 School of Epidemiology and Public Health, Faculty of Medicine, University of Ottawa, Ottawa, ON, Canada; 10 Department of Child and Adolescent Psychiatry, Charité Universitätsmedizin, Berlin, Germany; 11 Hampshire and Isle of Wight NHS Foundation Trust, Southampton, UK; 12 Clinical and Experimental Sciences (CNS and Psychiatry), Faculty of Medicine, University of Southampton, Southampton, UK; 13 Hassenfeld Children’s Hospital at NYU Langone, New York University Child Study Center, New York City, New York, USA; 14 DiMePRe-J-Department of Precision and Regenerative Medicine-Jonic Area, University of Bari “Aldo Moro”, Bari, Italy

**Keywords:** Child & adolescent psychiatry

## Abstract

**Background:**

Understanding the relationship between mental and physical health conditions is crucial for developing comprehensive healthcare strategies. The putative existence of a general disease factor (*d-factor*) that underlies the vulnerability to both physical and mental conditions could have important implications for our approach to health assessment and treatment.

**Objective:**

To investigate the presence and characteristics of a general *d-factor* in children and adolescents.

**Methods:**

This Swedish registry-based cross-sectional study included children and adolescents born between 1996 and 2003 with follow-up until 2013. We extracted data on 25 mental and physical health conditions according to the ICD-10 system. To determine the optimal dimensional structure of these conditions, several competing measurement models were tested, including correlated factors, one factor, various bifactor specifications and bifactor exploratory structural equation modelling (ESEM).

**Findings:**

The study cohort included 776 667 individuals (mean age 13.96 years, IQR=11.96–16.04; 51% male). The bifactor ESEM model, including a general *d-factor* and specific mental and physical health factors, provided the best fit to the data compared to alternative models (Comparative Fit Index=0.971, Tucker-Lewis Index=0.962, root mean square error of approximation=0.007 (0.007–0.007)). The *d-factor* accounted for substantial variance (ω_h_=0.582, explained common variance (ECV)=0.498), while specific mental (ω_hs_=0.377, ECV=0.373) and physical (ω_hs_=0.423; ECV=0.130) factors also indicated additional significant unique contributions.

**Conclusions:**

This study provided evidence for a multidimensional structure of health in children and adolescents, characterised by a general *d-factor* underlying both mental and physical conditions, alongside distinct domain-specific factors. These findings have important implications for clinical practice, providing evidence that suggests the need for more integrated approaches to health assessment and treatment that consider the interconnectedness of mental and physical health.

**WHAT IS ALREADY KNOWN ON THIS TOPIC**

- Does a general *d-factor* underlie both mental and physical health conditions in children and adolescents?

**WHAT THIS STUDY ADDS**

- Using Swedish nationwide registry data, we demonstrated that a bifactor exploratory structural equation model best captures the complex interplay between mental and physical health conditions. The general *d-factor* accounted for substantial variance ($\omega_{h}$=0.582, explained common variance (ECV)=0.498), while specific mental and physical health factors contributed significant unique variance ($\omega_{hs}$=0.377 and 0.423; ECV=0.373 and 0.130).

**HOW THIS STUDY MIGHT AFFECT RESEARCH, PRACTICE OR POLICY**

- Our findings revealed a multidimensional structure of health in children and adolescents, characterised by a general factor underlying both mental and physical conditions alongside distinct domain-specific factors. This study provides evidence that challenges the artificial split between physical and mental illness, calling for more integrated models of health assessment, prevention and treatment in early in life.

## Background

 The relationship between mental and physical conditions is an emerging area of research with important clinical and public healthcare implications. In relation to mental conditions, a general psychopathology factor, referred to as the *p-factor,*^1^,^3^ has been proposed to represent an individual’s overall propensity towards developing any form of psychopathology along a single dimension of severity.^1^ It emerged from modelling the structure of mental disorders, where instead of being distinct categories, disorders show substantial correlations and overlap with one another. The *p-factor* captures this shared variance across all psychopathological symptoms and disorders. Higher *p-factor* scores are associated with negative outcomes such as greater life impairment, compromised brain integrity and worse developmental trajectories, suggesting a unifying dimension that cuts across traditional diagnostic boundaries.^1^

While the *p-factor* represents a shared vulnerability across mental disorders, increasing evidence suggests that comorbidity extends beyond psychiatric conditions to physical health problems as well.^4 5^ Transdiagnostic associations have been reported across a wide range of mental and physical disorders,^6^ such as significant relationships between depression and diabetes,^7^ attention-deficit/hyperactivity disorder (ADHD) with depression, diabetes and other somatic conditions,^4 8 9^ or anorexia nervosa with inflammatory bowel diseases (IBD).^10^ This raises the possibility of an even broader disease liability factor, labelled the *d-factor*, reflecting a general propensity to both mental and physical health problems.^11^ The demonstration of a *d-factor* would support a conceptual shift from categorical differentiation between mental and physical illness towards viewing health problems dimensionally along an underlying continuum. It could also motivate an integrated approach to studying shared aetiological pathways and developing transdiagnostic interventions that cut across mind–body separations.^12^

Initial evidence for the existence of this hypothetical *d-factor* in adults has been reported in a recent study using data from the 1970 British Cohort Study (BCS).^13^ That study found that a bifactor model including both a general disease factor and more specific psychopathology and physical illness factors provided the best fit for explaining the covariance across diverse mental and medical conditions assessed in middle adulthood. However, the reliance on self-reported data for assessing mental and physical disorders, which can be subjected to recall and reporting biases, rather than clinical evaluations or medical records, was a limitation of the study. Additionally, the 1970 BCS cohort suffers from non-random attrition and healthy volunteer biases,^14^ limiting its representativeness. Furthermore, while that study focused on adults, it is unclear whether the *d-factor* only emerges later in life as individuals accumulate disorders over time or whether it is also present in children.

Focusing on young populations is highly relevant as awareness of a common vulnerability to mental and physical conditions in youth could support earlier integrated assessments as well as interventions and preventive strategies, potentially altering the trajectory of health outcomes. Furthermore, focusing on young populations is critical, as identifying a potential d-factor early in life would suggest it may represent a fundamental aspect of developmental health trajectories, rather than merely an accumulation of disorders over a long lifespan. A recent study^15^ provided initial support for a *d-factor* in adolescents but was limited by a relatively small sample size (1120 participants) and restricted to adolescents aged 14–18, potentially reducing generalisability. Additionally, that study relied on self-reported diagnoses, which, while valuable for capturing subjective experience, may be less precise for establishing specific clinical diagnoses compared with clinical assessments. It also examined a limited range of mental and physical health conditions. Furthermore, methodologically, the study did not include additional metrics or sensitivity analyses to assess unidimensionality and address known limitations of traditional factor models including standard bifactor models (eg, imposing an artificially simple structure by fixing cross-loadings to zero).

The current study aimed to investigate the structural evidence for, and quantify the substantive contribution of, a general *d-factor* in a population-based study based on Swedish registers, addressing the limitations of the current literature. We analysed a large nationwide multidecade cohort, spanning childhood through adolescence including clinically established rather than self-reported diagnoses. We aimed to model the structure of mental and physical conditions to determine whether the covariation is partly explained by a single overarching dimension reflecting a general propensity for illness. This approach allowed us to investigate whether the *d-factor* is a developmental phenomenon that emerges early in life or if it is indeed a result of cumulative health issues over time.

Based on the *p-factor* literature and preliminary evidence for a *d-factor*, we hypothesised that (H_1_) A factor model incorporating both a general *d-factor* and two specific latent factors (mental health and physical health) would provide a better fit to the data compared with models assuming only specific factors (correlated factors) or a single undifferentiated factor (one-factor model); (H_2_) specifically, a bifactor ESEM model, allowing for both a general *d-factor* and specific mental/physical factors with realistic cross-loadings, would represent the optimal structure; (H_3_) the general *d-factor* would account for a substantial portion of the common variance across conditions.

## Methods

### Study population

We conducted a registry-based cohort study drawing data from multiple Swedish national registers, including the total population register, the National Patient Register (NPR) and the Cause of Death Register. The study included all individuals born in Sweden between 1996 and 2003 with follow-up until 2013. Medical diagnoses in the NPR were coded using the International Classification of Diseases, 10th revision (ICD-10). The register provides comprehensive coverage of specialised healthcare, including all inpatient care since 1987 and outpatient services since 2001.

### Measures

Mental and physical health diagnoses, assessed until the end of follow-up (31 December 2013), were extracted from the NPR, and were selected based on relevance to children and adolescents, and availability in Swedish registers. These conditions represent a diverse range of conditions and disease mechanisms spanning different physiological and psychological systems (eg, neurological, metabolic, respiratory, inflammatory, neurodevelopmental, emotional, behavioural). This diversity is crucial for testing the hypothesis of a general disease factor that potentially cuts across these distinct domains and underlying mechanisms. Selected mental and neurodevelopmental conditions included ADHD, anxiety disorder, autism spectrum disorder (ASD), bipolar disorder, conduct disorder, eating disorders, intellectual disability (ID), learning and language disorders, major depressive disorder, obsessive-compulsive disorder (OCD) post-traumatic stress disorder (PTSD) and substance use disorder (SUD). These conditions typically emerge during childhood and adolescence. Physical health conditions included cardiovascular disease (such as congenital malformations of the circulatory system and cardiomyopathies), chronic respiratory diseases (including conditions such as chronic bronchitis and asthma), diabetes, eczema, epilepsy, hearing impairment, IBD, migraine, obesity, psoriasis, sleep disorders and visual impairment. ICD-10 codes used to identify each condition are reported in online supplemental table S1.

### Statistical analysis

To determine the latent structure underlying the covariation across mental and physical health conditions, seven factor models were tested within the structural equation modelling (SEM) framework: a correlated factors model, a one-factor model, a bifactor model with a general *d-factor*, two S-1 bifactor models with mental and physical health factors as reference respectively, an S·I-1 bifactor model, an exploratory structural equation model (ESEM)^16^ and a bifactor ESEM (all bifactor models assumed orthogonality, except the S·I-1). To assess model fit, we considered improvements in Comparative Fit Index (CFI) of 0.005–0.010 and concurrent decreases in root mean square error of approximation (RMSEA) of 0.010–0.015 as meaningful.^17^ Detailed descriptions of each model specification are provided in online supplemental material.

Models were estimated using Weighted Least Squares Mean and Variance (WLSMV)-adjusted estimation to handle binary diagnostic variables. Model fit was assessed using standard criteria: CFI and Tucker-Lewis Index (TLI) >0.95 and RMSEA <0.06 indicating good fit.^18^ In addition, we evaluated three specific indices that assess the strength and interpretation of the bifactor model.^19^ First, the explained common variance (ECV),^20^ which quantifies the proportion of variance in the observed variables attributable to the general factor. Higher ECV values indicate a strong and unidimensional general factor, whereas lower values suggest greater importance of the specific factors.^21^ Second, the percentage of uncontaminated correlations (PUC), which estimates the proportion of correlations among observed variables not influenced by the general factor. Higher PUC values suggest that the specific factors are distinct and meaningful, while lower values suggest that the general factor may be driving the relationship among the observed variables. Finally, we evaluated model-based reliability indices, specifically omega hierarchical ($\omega_{h}$) and omega subscale ($\omega_{s}$).^21^ These indices estimate the proportion of reliable variance in the observed variables that is accounted for by the general and specific factors, respectively. Together, these indices provide information on the relative strength and importance of the general and specific factors in the bifactor model. Reise *et al*^22^ suggest that when PUC values are lower than 0.80, general ECV values greater than 0.60 and $\omega_{h}$ greater than 0.70 for the general factor would support interpreting the instrument as primarily unidimensional, despite some multidimensionality. Construct replicability was assessed using the H-index,^23^ which indicates the stability of the latent variables across studies and how well-defined the latent constructs are. H-values between 0.70 nd 0.79 indicate acceptable construct replicability, while values above 0.80 suggest good replicability.

To assess measurement invariance across men and women, we conducted a multigroup confirmatory factor analysis using the WLSMV estimator. We tested two levels of invariance: configural and scalar. Configural invariance examines whether the same factor structure holds across groups, while scalar invariance tests whether item thresholds and factor loadings are equivalent across groups. Given the use of binary items and the WLSMV estimator, we did not test for metric invariance separately, as scalar invariance directly follows configural invariance in this context.^24^ Data management and statistical analyses were performed using SAS V.9.4 and Mplus^24^ V.8.3, respectively. We followed the Strengthening the Reporting of Observational Studies in Epidemiology reporting guideline.^25^

### Sensitivity analyses

While our primary model divided diseases into broad mental and physical health factors, we also examined a model that further subdivided the mental health factor into three components: internalising (anxiety, depression, OCD, PTSD, eating disorders), externalising (ADHD (as traditionally considered), conduct disorder, SUD), other mental health factors (bipolar disorder, ASD, ID, learning/language disorder (LLD), motor/TIC disorders, reflecting significant neurodevelopmental or neuropsychiatric conditions not clearly fitting internalising/externalising dimensions in youth), while retaining the physical health. We fitted several models to this four-factor structure including correlated factors, standard bifactor, S-1 bifactor, S·I-1 bifactor, bifactor ESEM and hierarchical models. For a detailed explanation for each model, see online supplemental material.

### Findings

The study cohort included 776 667 individuals with a median age of 13.96 (IQR=11.96–16.04; range=10–18) years and 398 348 (51%) were male. The most common health conditions were hearing (18%) and visual impairment (15%), ADHD (3.8%), ASD (1.6%) and anxiety disorder (1.6%). Table 1 summarises the cohort characteristics.

**Table 1:** Cohort characteristics

Characteristic	n (%)
N	776 667
*Sex*	
Males	398 348 (51)
Females	378 319 (49)
Age, median (IQR)	13.96 (11.96, 16.04)
Mental conditions	
Anxiety	12 808 (1.6)
Attention-deficit/hyperactivity disorder	29 405 (3.8)
Autism spectrum disorder	12 328 (1.6)
Bipolar disorder	474 (<0.1)
Conduct disorder	3663 (0.5)
Depression	8138 (1.0)
Eating disorder	3227 (0.4)
Intellectual disability	6962 (0.9)
Learning/language disorder	7267 (0.9)
Motor/TIC disorder	6392 (0.8)
Obsessive-compulsive disorder	2363 (0.3)
Post-traumatic stress disorder	1009 (0.1)
Substance use disorder	4219 (0.5)
Physical conditions	
Cardiovascular disease	13 979 (1.8)
Chronic respiratory diseases	85 268 (11)
Diabetes	4779 (0.6)
Eczema	17 059 (2.2)
Epilepsy	8139 (1.0)
Hearing impairment	140 497 (18)
Inflammatory bowel disease	16 046 (2.1)
Migraine	11 100 (1.4)
Obesity	14 587 (1.9)
Psoriasis	3034 (0.4)
Sleep disorders	10 049 (1.3)
Visual impairment	115 320 (15)

We compared several structural equation models to determine the best-fitting representation of the relationship between physical and mental health factors. Table 2 presents the fit indices for each model tested. The bifactor model with physical and mental health factors indicated the best fit to the data (χ²=15 133.14, CFI=0.955, TLI=0.946, RMSEA=0.009 (90% CI 0.009 to 0.009), SRMR=0.065). This model outperformed all tested models, with only the bifactor ESEM showing better fit (χ²= 9759.44, CFI=0.971, TLI=0.962, RMSEA=0.007 (90% CI 0.007 to 0.007), SRMR=0.054). Table 3 presents factor loadings for each item and latent factor (factor loadings for other models are shown in online supplemental tables S2–S4; tetrachoric correlations are presented in online supplemental table S5).

**Table 2:** Model fit statistics for the different factor model solutions

	Model	χ^2^	df	CFI	TLI	RMSEA (90% CI)	SRMR
Main analysis	Correlated factors	31 265.854	274	0.863	0.850	0.015 (0.015 to 0.015)	0.110
Main analysis	One-factor	40 472.285	275	0.823	0.807	0.017 (0.017 to 0.018)	0.117
Main analysis	Bifactor	15 133.143	250	0.955	0.946	0.009 (0.009 to 0.009)	0.065
Main analysis	S-1 bifactor (ref=mental)	31 995.248	262	0.905	0.891	0.012 (0.012 to 0.013)	0.083
Main analysis	S-1 bifactor (ref=physical)	33 259.124	263	0.901	0.887	0.013 (0.013 to 0.013)	0.108
Main analysis	S·I–1 bifactor	16 208.447	251	0.952	0.943	0.009 (0.009 to 0.009)	0.066
Main analysis	ESEM	23 874.961	251	0.929	0.915	0.011 (0.011 to 0.011)	0.075
Main analysis	Bifactor ESEM	9759.436	228	0.971	0.962	0.007 (0.007 to 0.007)	0.054
Sensitivity analysis*	Correlated factors	29 433.973	269	0.912	0.902	0.012 (0.012 to 0.012)	0.091
Sensitivity analysis*	Bifactor	15 950.467	248	0.953	0.943	0.009 (0.009 to 0.009)	0.071
Sensitivity analysis*	S-1 bifactor	19 763.744	254	0.941	0.931	0.010 (0.010 to 0.010)	0.087
Sensitivity analysis*	S·I–1 bifactor	13 613.870	245	0.96	0.951	0.008 (0.008 to 0.008)	0.062
Sensitivity analysis*	Bifactor ESEM	NA	NA	NA	NA	NA	NA
Sensitivity analysis*	Hierarchical	31 264.207	271	0.907	0.897	0.012 (0.012 to 0.012)	0.094

* Analysis further separated the mental factor into three factors: internalising, externalising and other mental health.

ADHD, attention-deficit/hyperactivity disorder; CFI, Comparative Fit Index; df, degrees of freedom; ESEM, exploratory structural equation modelling; NA, no convergence; Ref, reference domain; RMSEA, root mean square error of approximation; SRMR, standardised root mean square residual; TLI, Tucker-Lewis Index.

**Table 3:** Standardised factor loadings with standard errors for each binary item into the one-factor solution, correlated factors and into the bifactor ESEM with three latent factors (*d-factor*, mental and physical factors)

Item	One-factor	Correlated factors	Bifactor ESEM (orthogonal)		
		r=0.56 (0.005)	D-factor	Mental	Physical
Anxiety	0.686 (0.004)	0.759 (0.003)	0.356 (0.012)	0.745 (0.007)	0.096 (0.007)
ADHD	0.736 (0.003)	0.707 (0.004)	0.735 (0.006)	0.337 (0.012)	−0.043 (0.005)
Autism spectrum disorder	0.774 (0.004)	0.715 (0.004)	0.817 (0.004)	0.182 (0.013)	−0.056 (0.006)
Bipolar disorder	0.658 (0.011)	0.395 (0.008)	0.451 (0.018)	0.550 (0.018)	−0.062 (0.022)
Conduct disorder	0.682 (0.006)	0.788 (0.004)	0.593 (0.009)	0.426 (0.013)	−0.095 (0.009)
Depression	0.697 (0.004)	0.669 (0.007)	0.274 (0.013)	0.786 (0.007)	0.065 (0.008)
Eating disorders	0.466 (0.008)	0.577 (0.011)	0.013 (0.015)	0.655 (0.009)	0.113 (0.012)
Intellectual disability	0.743 (0.005)	0.695 (0.006)	0.884 (0.006)	−0.318 (0.017)	0.094 (0.007)
Learning/language disorder	0.545 (0.006)	0.485 (0.008)	0.631 (0.006)	−0.130 (0.013)	0.008 (0.007)
Motor/TIC disorder	0.656 (0.006)	0.747 (0.005)	0.678 (0.005)	0.170 (0.013)	0.026 (0.008)
Obsessive-compulsive disorder	0.651 (0.007)	0.557 (0.007)	0.394 (0.013)	0.681 (0.009)	0.029 (0.011)
Post-traumatic stress disorder	0.561 (0.011)	0.666 (0.011)	0.192 (0.018)	0.634 (0.013)	−0.011 (0.018)
Substance use disorder	0.379 (0.008)	0.667 (0.006)	0.190 (0.011)	0.455 (0.009)	−0.037 (0.011)
Cardiovascular disease	0.203 (0.007)	0.266 (0.008)	0.164 (0.007)	0.009 (0.008)	0.211 (0.007)
Chronic respiratory diseases	0.298 (0.004)	0.423 (0.004)	0.169 (0.005)	−0.011 (0.005)	0.599 (0.005)
Diabetes	0.134 (0.009)	0.361 (0.004)	0.084 (0.011)	0.046 (0.013)	0.118 (0.011)
Eczema	0.200 (0.007)	0.289 (0.007)	0.094 (0.007)	0.001 (0.008)	0.349 (0.007)
Epilepsy	0.514 (0.006)	0.197 (0.013)	0.577 (0.007)	−0.207 (0.006)	0.106 (0.009)
Hearing impairment	0.257 (0.004)	0.174 (0.011)	0.208 (0.004)	−0.076 (0.005)	0.373 (0.004)
Inflammatory bowel disease	0.274 (0.007)	0.621 (0.008)	0.134 (0.007)	0.028 (0.008)	0.424 (0.007)
Migraine	0.195 (0.007)	0.261 (0.008)	0.101 (0.008)	0.091 (0.009)	0.220 (0.008)
Obesity	0.323 (0.006)	0.387 (0.007)	0.241 (0.006)	0.086 (0.008)	0.285 (0.007)
Psoriasis	0.141 (0.012)	0.409 (0.007)	0.045 (0.014)	0.058 (0.015)	0.232 (0.013)
Sleep disorders	0.522 (0.006)	0.444 (0.004)	0.396 (0.008)	0.301 (0.007)	0.234 (0.008)
Visual impairment	0.316 (0.004)	0.642 (0.007)	0.295 (0.004)	−0.082 (0.005)	0.307 (0.004)

ADHD, attention-deficit/hyperactivity disorder; ESEM, exploratory structural equation modelling.

The results of the measurement invariance tests for sex (males vs females) are presented in online supplemental table S6. The configural model showed good fit, indicating that the same factor structure holds for both men and women. The scalar model, which constrained item thresholds and factor loadings to be equal across groups, also showed good fit. The change in fit indices between the configural and scalar models was minimal (ΔCFI=−0.001, ΔRMSEA=0, ΔSRMR=0.003), suggesting that scalar invariance was achieved. These results support the conclusion that the bifactor ESEM model is invariant across men and women, allowing for meaningful comparisons of latent means and variances between these groups.

We also evaluated more complex models that further divided the factors into internalising, externalising, other mental health factors and physical health. Among these, the bifactor model again showed the best fit, followed closely by the S-1 bifactor model. The hierarchical model and correlated factors model for these more differentiated factors showed poorer fit (table 2).

**Figure 1:**
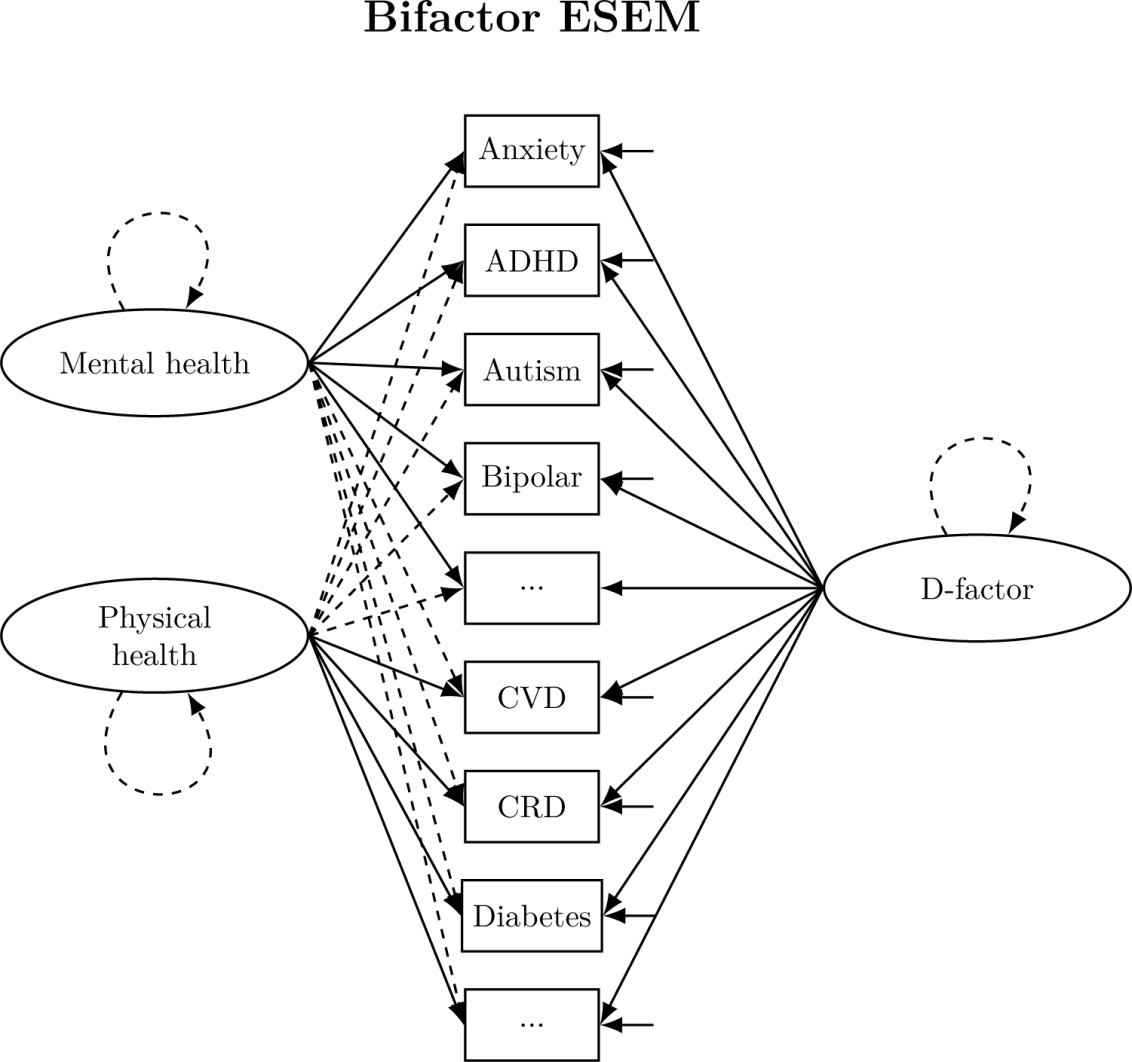
Representation of the bifactor ESEM model. Broken arrows represent cross-loadings. Factor variances fixed to 1. ADHD, attention-deficit/hyperactivity disorder; CRD, chronic respiratory diseases; CVD, cardiovascular disease; ESEM, exploratory structural equation modelling.

Given that the bifactor model ESEM with physical and mental health factors displayed the best overall fit (see figure 1 for a representation of the model), we focused our subsequent analyses on this model. The bifactor ESEM model revealed a general *d-factor* underlying both physical and mental health conditions, along with specific factors for mental and physical health. The ECV indicated that the *d-factor* accounted for 49.8% of the common variance, while mental and physical factors accounted for 37.3% and 13.0%, respectively. The omega hierarchical coefficient ($\omega_{h}$) for the general factor was 0.582, with the relative omega indicating that 66.2% of the reliable variance was attributable to the *d-factor*. The specific factors indicated meaningful unique contributions, with $\omega_{h}$ subscale values of 0.377 and 0.423 for the mental and physical factors, respectively. Construct replicability was strong for both the *d-factor* (H=0.913) and mental factor (H=0.865), while the physical factor showed moderate replicability (H=0.610). Following Reise *et al*^22^ suggestions, with a PUC of 0.520, general ECV of 0.498 and *d-factor* $\omega_{h}$=0.582, all three values fall short of these benchmarks, supporting a multidimensional structure rather than a primarily unidimensional one.

## Discussion

This study examined the structure of mental and physical health conditions in a large cohort of 776 667 children and adolescents with a median age of 14 years. Our primary aim was to investigate the structural evidence for and quantify the contribution of a general *d-factor* that might underlie both mental and physical health domains in children and adolescents, relative to specific health factors. Our results provided evidence supporting a general *d-factor* alongside specific mental and physical health factors. The bifactor ESEM model, which included these three components, showed superior fit compared with several alternative models. The better fit of the bifactor ESEM over the bifactor model indicated that allowing for cross-loadings while maintaining the bifactor structure best represents the underlying structure of health and illness in our data.

In simpler terms, our findings suggest that there is an underlying vulnerability (the *d-factor*) that increases an individual’s risk for developing both mental health problems (eg, anxiety or ADHD) and physical health problems (eg, diabetes). However, this general vulnerability represents only part of the complete picture. There are also specific factors related only to mental health, and only to physical health, that contribute uniquely to an individual’s health profile. This highlights the interconnectedness of mind and body from an early age, while also recognising the distinct nature of different health domains.

With a PUC of 0.520, general ECV of 0.498 and $\omega_{h}$ of 0.582, our results supported a multidimensional structure.^22^ Several indicators supported the relevance of the general *d-factor*. The general *d-factor* accounted for a substantial portion of the total variance and common variance across all items, indicating its importance in understanding overall health status. The construct replicability of the general factor was excellent (H=0.913), well above the 0.80 threshold for a well-defined latent variable,^23^ suggesting a stable and reliable dimension. The relative omega of 0.662 indicated that the general factor accounts for nearly two-thirds of the reliable variance. These values are particularly noteworthy given our study’s unique inclusion of both mental and physical health conditions, unlike traditional p-factor studies focusing solely on psychopathology. The specific factors for mental health and physical health also demonstrated significant unique contributions, particularly in terms of reliability.

Overall, our findings suggest that while there is a common underlying factor influencing both mental and physical health, there are also distinct aspects of mental and physical well-being that cannot be fully captured by a single general factor. This would support theoretical models proposing shared underlying mechanisms in health and disease,^26 27^ while acknowledging domain-specific variation. Both the general *d-factor* and specific factors appear to be meaningful constructs that warrant consideration in research and clinical practice. It is noteworthy that in the bifactor ESEM model, the standardised loadings of the mental health conditions onto the *d-factor* were generally higher than those for the physical health conditions. While the *d-factor* clearly captures variance across both domains, this pattern might suggest several possibilities. For example, the shared vulnerability pathways captured by ‘*d*’ manifest more strongly or are measured more reliably via the mental health indicators in this youth cohort. Alternatively, it could reflect referral or diagnostic practices in the specialised care system captured by the registers. Importantly, despite variable loading magnitudes, the model suggests a general factor influencing both mental and physical domains, arguing against a complete separation. Future research could explore whether this loading pattern differs in older cohorts or using different health indicators.

It is important to address a common misconception that the *d-factor* concept is simply a rebranding of multimorbidity. While both concepts deal with the co-occurrence of multiple health conditions, they differ significantly in their statistical approach and implications. Multimorbidity typically refers to the presence of two or more chronic conditions in an individual, often assessed through simple counts or pairwise associations. In contrast, the *d-factor* represents a latent construct that accounts for shared variance across a wide range of health conditions, captured through more sophisticated statistical techniques like bifactor modelling. This distinction is not merely academic. The *d-factor* approach suggests a common underlying vulnerability that increases risk across diverse health domains, whereas multimorbidity does not necessarily imply such a shared aetiology. In practice, this could lead to different approaches in prevention and treatment. A *d-factor* perspective might encourage more holistic, person-centred interventions targeting general health resilience, while a multimorbidity approach might focus more on managing specific disease combinations. However, more research is needed to fully elucidate the practical implications of these conceptual and statistical differences.

The identification of a general *d-factor* has several important implications. First, it suggests that there may be common underlying mechanisms or risk factors that influence both mental and physical health. This finding aligns with growing evidence of the interconnectedness of mind and body in health and disease processes.^28^ Second, the existence of a *d-factor* may help explain the high comorbidity often observed between mental and physical health conditions^4 5^ as well as the tendency for health problems to cluster within individuals.

Our findings align with and extend recent research on the concept of a general *d-factor,* preliminarily tested by Brandt *et al* in adults.^13^ Focusing on a younger cohort, we found evidence that this structure is evident earlier in life, suggesting that the *d-factor* may be a fundamental aspect of health that emerges relatively early and persists over time. Furthermore, our findings substantially extend the initial evidence for a *d-factor* reported by Stevanovic *et al*^15^ in Serbian adolescents, addressing key methodological limitations by employing more rigorous statistical approaches and analysing data from a comprehensive nationwide cohort of 776 667 Swedish youth, including both children and adolescents. Our use of national health registers with ICD-10 diagnoses provided clinically validated data, offering greater diagnostic precision than self-reported conditions.

Notably, ID and LLDs loaded negatively on the specific mental factor, contrasting with their positive loadings on the general d-factor. This likely reflects the bifactor model structure applied to complex comorbidities. The specific factor isolates residual mental variance (after accounting for the d-factor) but simplifies it into a single dimension largely defined by conditions loading most strongly on it (eg, internalising/externalising disorders). Because ID/LLD relate less strongly to this specific residual cluster, their negative loading indicates that, conditional on the d-factor, they are not associated with higher levels of this particular dimension. This underscores the need to consider model structure when interpreting specific factor loadings and suggests future research might benefit from models with more refined specific mental factors.

From a research and clinical/public health perspective, our results highlight the potential value of holistic approaches to health assessment and treatment in children and adolescents. While specialised care for specific mental or physical health conditions remains crucial, practitioners should be aware of the potential for broader health impacts beyond their immediate domain of focus. It also supports the implementation of preventive health measures that might yield improvements across both mental and physical health domains. For instance, in chronic disease management, treatment plans could routinely incorporate elements addressing both domains, recognising the potential for wide-ranging effects of interventions. Longitudinal patient monitoring, which is crucial in the developmental period, could adopt a more holistic approach, tracking indicators of both mental and physical health over time to detect early signs of comorbid condition development. At a systemic level, these findings support health policies that promote the integration of children and adolescent mental and physical health services, potentially leading to more efficient resource allocation and improved overall health outcomes. Moreover, these findings support the integrated model of care that should be encouraged in Consultation-Liaison psychiatry with children and adolescents, highlighting the need for mental health expertise within general medical settings and vice versa, given the shared underlying vulnerability (*d-factor*) across physical and mental conditions frequently encountered in Consultation-Liaison work. From a research standpoint, the *d-factor* concept could serve as a basis for developing more sophisticated risk stratification tools and inform transdiagnostic treatment approaches that target common underlying mechanisms.

Overall, we strengthened methodological rigour through advanced modelling techniques, incorporating crucial bifactor metrics and comprehensive sensitivity analyses. Unlike previous studies that relied exclusively on model fit indices, our study employed essential bifactor-specific indices ($\omega_{h}$, ECV, H-index) to evaluate factor reliability and replicability. Furthermore, our use of bifactor ESEM modelling allowed for cross-loadings, providing a more realistic representation of health conditions compared with traditional CFA approaches that can artificially inflate factor correlations through imposed simple structure. However, some limitations of this study should be noted. First, while our sample size was large, the relatively young age of the cohort may limit generalisability to older populations. Health conditions and their inter-relationships may evolve differently across the lifespan, requiring further research in more diverse age groups. Second, our study relied on medical records, which may be subject to misclassification. Furthermore, our reliance on specialised care data resulted in prevalence estimates lower than those typically found in community-based studies,^29^ which capture milder or undiagnosed cases. This limitation may have introduced a selection bias towards more severe or complex cases, potentially affecting the generalisability, but, crucially, not the accuracy of our findings. Future studies could benefit from incorporating multiple data sources, including objective health measures and longitudinal designs, to better understand the stability and predictive validity of the *d-factor* over time. Future studies should explore whether these findings are replicable in other populations. Third, the data follow-up concluded at the end of 2013. While this provides a comprehensive longitudinal picture up to that point for our birth cohorts, it is acknowledged that healthcare practices, treatments and potentially subtle aspects of diagnostic application may have evolved since then. However, given our focus on modelling the fundamental covariance structure among major, clinically diagnosed conditions based on ICD-10 in a very large sample, we argue that the identified latent structure, including the general *d-factor*, likely reflects relatively stable underlying patterns of comorbidity. Additionally, while our bifactor ESEM model showed good fit, the complexity of human health means that even this model is likely a simplification of the true underlying structure. Future research could explore more nuanced models, incorporating environmental, genetic or lifestyle factors that may influence the relationship between mental and physical health.

In conclusion, our study provides evidence for a multidimensional structure of health, characterised by a general *d-factor* that underlies both mental and physical health conditions, alongside specific factors for each domain. This finding has significant implications for how we conceptualise, assess and treat health conditions, encouraging a more integrated approach to healthcare. As we continue to unravel the complex interplay between mind and body, the *d-factor* concept offers a promising framework for advancing our understanding of human health and well-being. Future research should focus on replicating these findings in diverse populations, exploring the biological and environmental underpinnings of the *d-factor* and translating these insights into improved clinical practices and public health strategies.

## Supplementary material

10.1136/bmjment-2025-301592 online supplemental file 1

## Data Availability

Data may be obtained from a third party and are not publicly available.
