# Supplementary material for: General disease factor: evidence of a unifying dimension across mental and physical illness in children and adolescents
Source: BMJ Ment Health. 2025 Jun 3;28(1):e301592. doi: 10.1136/bmjment-2025-301592 (PMC12142111; doi:10.1136/bmjment-2025-301592)
Supplement: online supplemental file 1 [file bmjment-28-1-s001.docx]

**Supplementary material for: A General Disease Factor: Evidence of a Unifying Dimension Across Mental and Physical Illness in Children and Adolescents**

**Contents**

[Methods 2](#_Toc198049378)

[Table S1. ICD codes used to define each psychiatric/medical condition 8](#_Toc198049379)

[Table S2. Factor loadings for the correlated-factor models further dividing mental health into internalizing, externalizing and others. 9](#_Toc198049380)

[Table S3. Factor loadings for the bifactor and Bifactor (S·I – 1) models further dividing mental health into internalizing, externalizing and others. 10](#_Toc198049381)

[Table S4. Factor loadings for hierarchical models further dividing mental health into internalizing, externalizing and others. 12](#_Toc198049382)

[Table S5. Configural and scalar invariance 13](#_Toc198049383)

[Table S6. Tetrachoric correlations 14](#_Toc198049384)

[References 15](#_Toc198049385)

# Methods

The 13 models tested (8 in the main analyses and 5 in sensitivity analyses) represent different theoretical approaches to understanding the relationship between mental and physical health conditions. Each model employs different parameter constraints and factor structures to test specific hypotheses about the underlying architecture of comorbidity patterns.

***Correlated factors model***

The correlated factors model specifies separate but correlated factors for mental and physical conditions, assuming these are distinct but related constructs. This approach maintains theoretical independence while quantifying the strength of association between domains through factor correlations. The model estimates domain-specific factor loadings ($\lambda$) for each condition on its respective latent factor, with the correlation parameter ($\psi$) between factors providing a direct estimate of the strength of association between mental and physical health domains. This specification allows examination of domain-specific variance while acknowledging potential shared etiological mechanisms or mutually influencing processes between domains. The model's parsimony and interpretability make it particularly valuable for initial tests of domain distinctiveness.

***One-factor model (unidimensional model)***

The one-factor model takes a simpler approach by specifying a single general factor ($g$) that accounts for variance across all health conditions regardless of traditional classification. This model tests the hypothesis that there is a single underlying factor that influences both mental and physical conditions, suggesting no meaningful distinction between these domains. The model estimates a single set of factor loadings ($\lambda$) for all conditions, with the magnitude of these loadings indicating the strength of relationship between each condition and the general factor. This approach would be theoretically aligned with perspectives emphasizing common biological, psychological, or environmental determinants across all health conditions. The model serves as an important baseline for evaluating whether more complex factor structures represent significant improvements in fit.

***Bifactor model***

The bifactor model introduces a more complex structure incorporating both a general disease liability factor (*d-factor*), reflecting variance common to all conditions, and orthogonal specific factors ($S$) representing residual variance unique to the mental ($M$) and physical ($P$) health domains. This specification allows each condition to simultaneously load on both the general factor and its respective domain-specific factor, partitioning variance into general and specific components. The model estimates general factor loadings ($\lambda_{g}$) for all conditions and domain-specific loadings ($\lambda_{s}$) for mental and physical conditions on their respective specific factors, with orthogonality constraints ($cov(g,M)=cov(g,P)=cov(M,P)=0$) enforced between all factors. This approach enables quantification of both shared vulnerability across all conditions and domain-specific variance unexplained by the general factor, providing a more nuanced representation of comorbidity structures.

**S-1 bifactor model**

To address the limitations of the standard bifactor model under typical single-level sampling conditions with distinct domains, Eid et al. (2017)^1^ proposed alternative formulations. The $S-1$ bifactor model represents a more constrained version of the bifactor model where one specific factor (we tested both physical and mental health conditions) serves as reference and is ‘*absorbed’* into the general factor. The model maintains orthogonality between the general factor and the remaining specific factor but relaxes the constraint that reference group items cannot load on specific factors. This specification estimates general factor loadings ($\lambda_{g}$) for all conditions and specific factor loadings ($\lambda_{s}$) only for the non-reference domain, with the orthogonality constraint $cov(g,M)=0$ enforced. This model has one fewer specific factor than the number of domains ($K$ domains yield $K-1$ specific factors, hence "$S-1$"). This model is often recommended as a more parsimonious alternative to the standard bifactor model, as it relaxes the orthogonality assumption and helps avoid potential issues with factor over-extraction and improper solutions.^1,2^

**S·I-1 bifactor model**

The $S\cdot I-1$ bifactor model offers a less restrictive approach, allowing for a higher degree of heterogeneity in the factor structure.^1^ Similar to the $S-1$ model, this model defines the general factor using a single reference indicator from a reference domain. However, it defines specific (residual) factors for all domains, including the reference domain. The key distinction lies in how these specific factors are linked to indicators: the primary reference indicator ($Y_{11}$, whose true score defines $G$) does not load on any specific factor, including the specific factor for its own domain. The specific factor for the reference domain is defined based on the residual true score of a different indicator within that reference domain. Specific factors for non-reference domains are defined as residuals based on an indicator within that domain. This results in $S$ specific factors (where $S=K$, the number of domains) but only $I-1$ specific factor loadings (as the reference indicator $Y_{11}$ has no specific factor loading, hence "$S\cdot I-1$"). This model allows for modeling heterogeneity (specific variance) even within the reference domain, beyond what is captured by $G$. Like the $S-1$ model, $G$ is uncorrelated with specific factors, specific factors can be correlated, and specific factor means are zero. It can provide better fit than the $S-1$ model if the reference domain itself contains significant specific variance, or the reference indicator is not perfectly representative of the general factor construct.

***Bifactor Exploratory Structural Equation Model (ESEM)***

The bifactor ESEM^3^ integrates confirmatory bifactor modeling with exploratory factor analysis techniques through an orthogonal bifactor target rotation. Crucially, unlike traditional bifactor CFA models that constrain cross-loadings to zero, this specification allows for the estimation of small cross-loadings ($\lambda_{cross}$) between items and non-target specific factors while maintaining orthogonality constraints among all factors. The model estimates general factor loadings ($\lambda_{g}$) for all conditions, plus target specific factor loadings ($\lambda_{target}$) and non-target cross-loadings, with magnitudes of cross-loadings typically smaller than target loadings but not fixed at zero. This hybrid approach balances the theoretical structure of bifactor models with the empirical flexibility of allowing conditions to relate to both mental and physical domains simultaneously. The resulting factor structure often demonstrates superior fit indices compared to more constrained models while providing a more realistic representation of the complex relationships between health conditions that may not fit neatly into traditional taxonomic boundaries.

**Sensitivity analyses**

In sensitivity analyses, we implemented a hierarchical factor model to provide an alternative conceptualization of the relationship between mental and physical health conditions. This second-order factor structure specifies mental ($M$) and physical ($P$) condition factors as first-order latent variables that subsequently load onto a single higher-order general disease liability factor (*d-factor*). The hierarchical structure imposes a proportionality constraint on the covariances among first-order factors, as these relationships are fully mediated through the higher-order factor. In other words, the model assumes that the only reason the first-order factors ($M$ and $P$) are correlated is their shared dependence on the second-order factor ($G$). The covariance between $M$ and $P$ is therefore constrained to be the product of their respective loadings on $G$ multiplied by the variance of $G$ ($Cov(M,P) = \gamma M * \gamma P * Var(G)$). If first-order factors share variance due to sources other than the specified second-order factor, this constraint will be violated, leading to poor model fit.

Unlike bifactor models where each indicator loads directly on both general and specific factors, the hierarchical model channels the general factor's influence indirectly through the first-order factors, creating a more parsimonious factor structure with fewer estimated parameters. Statistically, this model involves two levels of factor loadings. First-order loadings ($\lambda_{1}$) link the observed conditions to their respective first-order factors ($M$ and $P$), similar to the correlated factors model. Second-order loadings ($\gamma$ or $\lambda_{2}$) then link the first-order factors ($M$ and $P$) to the overarching second-order factor ($G$). Crucially, the direct influence of the general factor ($G$) on the observed conditions is channeled entirely through the first-order factors. This contrasts sharply with the bifactor model, where observed variables load directly onto both the general and specific factors. The hierarchical model also estimates residual variances (disturbances, often denoted $\zeta$) for the first-order factors ($\zeta_{M}$, $\zeta_{P}$). These residuals represent the variance in each first-order factor that is not explained by the second-order general factor, capturing domain specificity at the factor level.

This hierarchical representation offers several theoretical and methodological advantages. First, it aligns with nested taxonomic systems common in medical classification, where specific conditions belong to broader categories that share etiological or phenomenological features. Second, it addresses concerns regarding bifactor models' potential over-extraction of general factor variance and tendency toward unstable solutions, particularly when the true factor structure is hierarchical rather than bifactor in nature. The hierarchical model imposes a more restrictive structure that often yields more stable parameter estimates across samples.

## Table S1. ICD codes used to define each psychiatric/medical condition

| **Condition** | **ICD-10** |
| --- | --- |
| Anxiety | F40-F42, F44-F45, F48 |
| Attention-deficit/hyperactivity disorder | F90 |
| Autism spectrum disorder | F84 |
| Bipolar disorder | F30, F31 |
| Cardiovascular disease | I00-I99 |
| Chronic respiratory diseases | J40-J47 |
| Conduct disorder | F91 |
| Depression | F32–34 |
| Diabetes | E10-E11 |
| Eating disorder | F50.0-F50.3, F50.9 |
| Eczema | L30 |
| Epilepsy | G40-G41 |
| Hearing impairment | H6 |
| Inflammatory bowel disease | K50-K52 |
| Intellectual disability | F70 -F73, F78, F79 |
| Learning/language disorder | F80, F81, F83, R48 |
| Migraine | G43 |
| Motor/TIC disorder | F82, F98.4, F95 |
| Obesity | E65-E66 |
| Obsessive-compulsive disorder | F42 |
| Post-traumatic stress disorder | F43.1 |
| Psoriasis | L40 |
| Sleep disorders | G47, F51 |
| Substance use disorder | F1 |
| Visual impairment | H1-H5 |

## Table S2. Factor loadings for the correlated-factor models further dividing mental health into internalizing, externalizing and others.

| **Correlated factors** | | |
| --- | --- | --- |
|  | **Parameter** | **b (SE)** |
| Intern.by | ANX | 0.895 (0.004) |
| Intern.by | DEP | 0.805 (0.005) |
| Intern.by | OCD | 0.868 (0.006) |
| Intern.by | PTSD | 0.640 (0.009) |
| Intern.by | EATING | 0.566 (0.007) |
| Extern.by | ADHD | 0.929 (0.005) |
| Extern.by | CONDUCT | 0.738 (0.005) |
| Extern.by | SUD | 0.432 (0.008) |
| Other.by | BIP | 0.715 (0.012) |
| Other.by | ASD | 0.836 (0.003) |
| Other.by | INT_DISAB | 0.80 (0.004) |
| Other.by | LEARN_LANG | 0.597 (0.005) |
| Other.by | MOTOR_TIC | 0.707 (0.005) |
| Physical.by | CVD | 0.273 (0.006) |
| Physical.by | EYES | 0.429 (0.003) |
| Physical.by | EARS | 0.388 (0.003) |
| Physical.by | ECZEMA | 0.303 (0.006) |
| Physical.by | PSORIASIS | 0.205 (0.011) |
| Physical.by | DIABETES | 0.154 (0.009) |
| Physical.by | EPILEP | 0.644 (0.006) |
| Physical.by | MIGRAINE | 0.245 (0.006) |
| Physical.by | IBS | 0.379 (0.006) |
| Physical.by | OBES | 0.391 (0.005) |
| Physical.by | CHRON_RESP | 0.461 (0.003) |
| Physical.by | SLEEP | 0.585 (0.006) |
| Intern.with | PHYSICAL | 0.311 (0.006) |
| Intern.with | EXTERN | 0.585 (0.006) |
| Intern.with | OTHER | 0.524 (0.006) |
| Other.with | EXTERN | 0.803 (0.006) |
| Physical.with | EXTERN | 0.404 (0.005) |
| Physical.with | OTHER | 0.663 (0.005) |

## Table S3. Factor loadings for the bifactor and Bifactor (S·I – 1) models further dividing mental health into internalizing, externalizing and others.

|  |  | **Bifactor** | **Bifactor (S·I – 1)** |
| --- | --- | --- | --- |
|  | **Parameter** | **b (SE)** | **b (SE)** |
| Intern.by | ANX | 0.69 (0.007) | 0.771 (0.006) |
| Intern.by | DEP | 0.566 (0.007) | 0.742 (0.007) |
| Intern.by | OCD | 0.648 (0.01) | 0.726 (0.009) |
| Intern.by | PTSD | 0.456 (0.016) | 0.601 (0.013) |
| Intern.by | EATING | 0.654 (0.012) | 0.656 (0.009) |
| Extern.by | ADHD | 0.593 (0.066) | 0.505 (0.01) |
| Extern.by | CONDUCT | 0.228 (0.026) | 0.661 (0.012) |
| Extern.by | SUD | -0.093 (0.021) | 0.6 (0.014) |
| Other.by | BIP | -0.261 (0.028) | 0.596 (0.023) |
| Other.by | ASD | 0.288 (0.008) | 0.218 (0.01) |
| Other.by | INT_DISAB | 0.838 (0.013) | -0.348 (0.018) |
| Other.by | LEARN_LANG | 0.422 (0.009) | -0.077 (0.011) |
| Other.by | MOTOR_TIC | 0.246 (0.01) | 0.203 (0.011) |
| Physical.by | CVD | 0.248 (0.007) | - |
| Physical.by | EYES | 0.405 (0.004) | 0.302 (0.004) |
| Physical.by | EARS | 0.431 (0.004) | 0.367 (0.004) |
| Physical.by | ECZEMA | 0.351 (0.006) | 0.335 (0.006) |
| Physical.by | PSORIASIS | 0.216 (0.011) | 0.202 (0.013) |
| Physical.by | DIABETES | 0.127 (0.01) | 0.097 (0.011) |
| Physical.by | EPILEP | 0.414 (0.008) | 0.082 (0.008) |
| Physical.by | MIGRAINE | 0.208 (0.007) | 0.176 (0.008) |
| Physical.by | IBS | 0.42 (0.006) | 0.404 (0.007) |
| Physical.by | OBES | 0.31 (0.006) | 0.256 (0.007) |
| Physical.by | CHRON_RESP | 0.566 (0.004) | 0.628 (0.006) |
| Physical.by | SLEEP | 0.241 (0.008) | 0.13 (0.008) |
| D.by | ADHD | 0.835 (0.004) | 0.692 (0.006) |
| D.by | ANX | 0.576 (0.005) | 0.406 (0.008) |
| D.by | DEP | 0.549 (0.005) | 0.348 (0.008) |
| D.by | SUD | 0.473 (0.01) | 0.141 (0.011) |
| D.by | ASD | 0.776 (0.004) | 0.783 (0.004) |
| D.by | OCD | 0.559 (0.008) | 0.41 (0.011) |
| D.by | PTSD | 0.441 (0.013) | 0.255 (0.015) |
| D.by | CONDUCT | 0.664 (0.007) | 0.472 (0.01) |
| D.by | EATING | 0.21 (0.011) | 0.072 (0.012) |
| D.by | INT_DISAB | 0.544 (0.006) | 0.952 (0.006) |
| D.by | LEARN_LANG | 0.453 (0.006) | 0.621 (0.006) |
| D.by | BIP | 0.777 (0.012) | 0.408 (0.018) |
| D.by | MOTOR_TIC | 0.658 (0.006) | 0.662 (0.006) |
| D.by | CVD | 0.107 (0.007) | 0.237 (0.007) |
| D.by | EYES | 0.154 (0.004) | 0.287 (0.004) |
| D.by | EARS | 0.075 (0.004) | 0.205 (0.004) |
| D.by | ECZEMA | 0.021 (0.007) | 0.114 (0.007) |
| D.by | PSORIASIS | 0.032 (0.014) | 0.083 (0.013) |
| D.by | DIABETES | 0.075 (0.011) | 0.106 (0.01) |
| D.by | EPILEP | 0.406 (0.007) | 0.562 (0.005) |
| D.by | MIGRAINE | 0.105 (0.008) | 0.151 (0.007) |
| D.by | IBS | 0.061 (0.007) | 0.162 (0.007) |
| D.by | OBES | 0.205 (0.007) | 0.278 (0.006) |
| D.by | CHRON_RESP | 0.053 (0.005) | 0.182 (0.005) |
| D.by | SLEEP | 0.475 (0.006) | 0.519 (0.006) |
| D.with | INTERN | 0 (0) | 0 (0) |
| D.with | EXTERN | 0 (0) | 0 (0) |
| D.with | OTHER | 0 (0) | 0 (0) |
| D.with | PHYSICAL | 0 (0) | 0 (0) |
| Intern.with | PHYSICAL | 0 (0) | 0.516 (0.01) |
| Intern.with | EXTERN | 0 (0) | 0.806 (0.025) |
| Intern.with | OTHER | 0 (0) | 0.978 (0.028) |
| Other.with | EXTERN | 0 (0) | -0.012 (0.009) |
| Physical.with | EXTERN | 0.169 (0.022) | -0.05 (0.01) |
| Physical.with | OTHER | 0.595 (0.011) | -0.132 (0.016) |

## Table S4. Factor loadings for hierarchical models further dividing mental health into internalizing, externalizing and others.

|  | **Parameter** | **b (SE)** |
| --- | --- | --- |
| Intern.by | ANX | 0.895 (0.004) |
| Intern.by | DEP | 0.805 (0.005) |
| Intern.by | OCD | 0.868 (0.006) |
| Intern.by | PTSD | 0.640 (0.009) |
| Intern.by | EATING | 0.566 (0.007) |
| Extern.by | ADHD | 0.929 (0.005) |
| Extern.by | CONDUCT | 0.738 (0.005) |
| Extern.by | SUD | 0.432 (0.008) |
| Other.by | BIP | 0.715 (0.012) |
| Other.by | ASD | 0.836 (0.003) |
| Other.by | INT_DISAB | 0.80 (0.004) |
| Other.by | LEARN_LANG | 0.597 (0.005) |
| Other.by | MOTOR_TIC | 0.707 (0.005) |
| Physical.by | CVD | 0.273 (0.006) |
| Physical.by | EYES | 0.429 (0.003) |
| Physical.by | EARS | 0.388 (0.003) |
| Physical.by | ECZEMA | 0.303 (0.006) |
| Physical.by | PSORIASIS | 0.205 (0.011) |
| Physical.by | DIABETES | 0.154 (0.009) |
| Physical.by | EPILEP | 0.644 (0.006) |
| Physical.by | MIGRAINE | 0.245 (0.006) |
| Physical.by | IBS | 0.379 (0.006) |
| Physical.by | OBES | 0.391 (0.005) |
| Physical.by | CHRON_RESP | 0.461 (0.003) |
| Physical.by | SLEEP | 0.585 (0.006) |
| Intern.with | PHYSICAL | 0.311 (0.006) |
| Intern.with | EXTERN | 0.585 (0.006) |
| Intern.with | OTHER | 0.524 (0.006) |
| Other.with | EXTERN | 0.803 (0.006) |
| Physical.with | EXTERN | 0.404 (0.005) |
| Physical.with | OTHER | 0.663 (0.005) |

## Table S5. Configural and scalar invariance

|  | **Model** | **χ^2^** | ***df*** | **CFI** | **TLI** | **SRMR** | **RMSEA** | **90% CI** | **ΔCFI** | **ΔRMSEA** | **ΔSRMR** |
| --- | --- | --- | --- | --- | --- | --- | --- | --- | --- | --- | --- |
| Sex: males vs females |  |  |  |  |  |  |  |  |  |  |  |
| Bifactor | Configural | 8941.99 | 456 | 0.974 | 0.966 | 0.055 | 0.007 | 0.007-0.007 | - | - | - |
|  | Scalar | 9275.88 | 519 | 0.973 | 0.969 | 0.058 | 0.007 | 0.006-0.007 | -0.001 | 0 | 0.003 |

## Table S6. Tetrachoric correlations

|  | ANX | DEP | OCD | PTSD | EATING | ADHD | COND | SUD | BIP | ASD | INT  DISAB | LEARN  LANG | MOTOR  TIC | CVD | EYES | EARS | ECZEMA | PSORIASIS | DIAB | EPILEP | MIGRAINE | IBS | OBES | CHRON  RESP | SLEEP |
| --- | --- | --- | --- | --- | --- | --- | --- | --- | --- | --- | --- | --- | --- | --- | --- | --- | --- | --- | --- | --- | --- | --- | --- | --- | --- |
| ANX | 1 |  |  |  |  |  |  |  |  |  |  |  |  |  |  |  |  |  |  |  |  |  |  |  |  |
| DEP | 0.44 | 1 |  |  |  |  |  |  |  |  |  |  |  |  |  |  |  |  |  |  |  |  |  |  |  |
| OCD | 0.45 | 0.67 | 1 |  |  |  |  |  |  |  |  |  |  |  |  |  |  |  |  |  |  |  |  |  |  |
| PTSD | 0.34 | 0.38 | 0.42 | 1 |  |  |  |  |  |  |  |  |  |  |  |  |  |  |  |  |  |  |  |  |  |
| EATING | 0.68 | 0.43 | 0.41 | 0.13 | 1 |  |  |  |  |  |  |  |  |  |  |  |  |  |  |  |  |  |  |  |  |
| ADHD | 0.41 | 0.94 | 0.48 | 0.19 | 0.46 | 1 |  |  |  |  |  |  |  |  |  |  |  |  |  |  |  |  |  |  |  |
| CONDUCT | 0.32 | 0.50 | 0.62 | 0.35 | 0.15 | 0.34 | 1 |  |  |  |  |  |  |  |  |  |  |  |  |  |  |  |  |  |  |
| SUD | 0.69 | 0.42 | 0.44 | 0.41 | 0.46 | 0.36 | 0.41 | 1 |  |  |  |  |  |  |  |  |  |  |  |  |  |  |  |  |  |
| BIP | 0.11 | 0.50 | 0.60 | 0.24 | 0.14 | 0.43 | 0.32 | 0.12 | 1 |  |  |  |  |  |  |  |  |  |  |  |  |  |  |  |  |
| ASD | 0.47 | 0.18 | 0.08 | 0.04 | 0.67 | 0.19 | 0.10 | 0.25 | -0.01 | 1 |  |  |  |  |  |  |  |  |  |  |  |  |  |  |  |
| INT  DISAB | 0.42 | 0.13 | 0.06 | 0.00 | 0.50 | 0.13 | 0.06 | 0.19 | 0.00 | 0.59 | 1 |  |  |  |  |  |  |  |  |  |  |  |  |  |  |
| LEARN  LANG | 0.56 | 0.52 | 0.59 | 0.35 | 0.44 | 0.44 | 0.44 | 0.55 | 0.35 | 0.21 | 0.14 | 1 |  |  |  |  |  |  |  |  |  |  |  |  |  |
| MOTOR  TIC | 0.58 | 0.38 | 0.23 | 0.07 | 0.56 | 0.50 | 0.13 | 0.42 | 0.09 | 0.50 | 0.47 | 0.38 | 1 |  |  |  |  |  |  |  |  |  |  |  |  |
| CVD | 0.08 | 0.11 | 0.08 | 0.06 | 0.08 | 0.08 | 0.01 | 0.04 | 0.11 | 0.20 | 0.09 | 0.08 | 0.12 | 1 |  |  |  |  |  |  |  |  |  |  |  |
| EYES | 0.14 | 0.09 | 0.07 | 0.02 | 0.20 | 0.08 | 0.03 | 0.08 | 0.04 | 0.38 | 0.18 | 0.06 | 0.21 | 0.12 | 1 |  |  |  |  |  |  |  |  |  |  |
| EARS | 0.12 | 0.05 | 0.00 | -0.02 | 0.12 | 0.05 | -0.04 | 0.06 | -0.02 | 0.23 | 0.18 | 0.05 | 0.14 | 0.12 | 0.17 | 1 |  |  |  |  |  |  |  |  |  |
| ECZEMA | 0.05 | 0.07 | 0.01 | -0.01 | 0.07 | 0.07 | -0.03 | 0.04 | 0.04 | 0.11 | 0.05 | 0.01 | 0.07 | 0.08 | 0.13 | 0.15 | 1 |  |  |  |  |  |  |  |  |
| PSORIASIS | 0.04 | 0.06 | 0.08 | 0.10 | 0.06 | 0.04 | 0.00 | 0.03 | 0.06 | 0.06 | 0.04 | 0.08 | 0.05 | 0.07 | 0.06 | 0.05 | 0.35 | 1 |  |  |  |  |  |  |  |
| DIABETES | 0.06 | 0.07 | 0.09 | 0.11 | 0.07 | 0.02 | -0.05 | 0.03 | 0.04 | 0.06 | 0.04 | -0.04 | 0.03 | 0.08 | 0.06 | 0.06 | 0.07 | 0.07 | 1 |  |  |  |  |  |  |
| EPILEP | 0.26 | 0.14 | 0.06 | 0.02 | 0.41 | 0.09 | 0.09 | 0.16 | 0.00 | 0.65 | 0.34 | 0.07 | 0.33 | 0.20 | 0.23 | 0.16 | 0.10 | 0.06 | 0.10 | 1 |  |  |  |  |  |
| MIGRAINE | 0.07 | 0.14 | 0.14 | 0.07 | 0.06 | 0.05 | 0.08 | 0.04 | 0.10 | 0.01 | 0.01 | 0.09 | 0.08 | 0.12 | 0.13 | 0.06 | 0.06 | 0.05 | 0.07 | 0.19 | 1 |  |  |  |  |
| IBS | 0.11 | 0.10 | 0.07 | -0.01 | 0.12 | 0.06 | -0.01 | 0.09 | 0.07 | 0.13 | 0.09 | 0.07 | 0.11 | 0.12 | 0.15 | 0.17 | 0.17 | 0.07 | 0.09 | 0.10 | 0.12 | 1 |  |  |  |
| OBES | 0.21 | 0.15 | 0.18 | 0.05 | 0.21 | 0.07 | 0.06 | 0.17 | 0.03 | 0.23 | 0.13 | 0.13 | 0.16 | 0.10 | 0.12 | 0.14 | 0.13 | 0.11 | 0.12 | 0.12 | 0.09 | 0.11 | 1 |  |  |
| CHRON  RESP | 0.15 | 0.08 | 0.04 | 0.02 | 0.11 | 0.04 | 0.00 | 0.10 | 0.02 | 0.13 | 0.10 | 0.06 | 0.13 | 0.13 | 0.25 | 0.27 | 0.20 | 0.05 | 0.05 | 0.10 | 0.13 | 0.30 | 0.24 | 1 |  |
| SLEEP | 0.38 | 0.38 | 0.41 | 0.23 | 0.34 | 0.25 | 0.38 | 0.34 | 0.19 | 0.30 | 0.19 | 0.31 | 0.27 | 0.12 | 0.14 | 0.21 | 0.12 | 0.02 | 0.05 | 0.25 | 0.12 | 0.14 | 0.21 | 0.16 | 1 |

## References

1 Eid M, Geiser C, Koch T, Heene M. Anomalous results in G-factor models: Explanations and alternatives. *Psychol Methods* 2017; **22**: 541–62.

2 Heinrich M, Zagorscak P, Eid M, Knaevelsrud C. Giving G a Meaning: An Application of the Bifactor-(S-1) Approach to Realize a More Symptom-Oriented Modeling of the Beck Depression Inventory–II. *Assessment* 2020; **27**: 1429–47.

3 Asparouhov T, Muthén B. Exploratory Structural Equation Modeling. *Struct Equ Model Multidiscip J* 2009; **16**: 397–438.
